# Comparative study of cefixime and tetracycline as an evaluation policy driven by the antibiotic resistance crisis in Indonesia

**DOI:** 10.1038/s41598-021-98129-y

**Published:** 2021-09-16

**Authors:** Danni Ramdhani, Sri Agung Fitri Kusuma, Dede Sediana, A. P. Hilarius Bima, Ika Khumairoh

**Affiliations:** 1 Department of Pharmaceutical Analysis and Medicinal Chemistry, Faculty of Pharmacy, Padjadjaran University, Sumedang, Indonesia; 2 Department of Biology Pharmacy, Faculty of Pharmacy, Padjadjaran University, Sumedang, Indonesia; 3 Pharmaceutical Division, Tasikmalaya City Health Office, Tasikmalaya, West Java Province Indonesia

**Keywords:** Microbiology, Antimicrobials, Microbial communities, Policy and public health in microbiology

## Abstract

Antibiotic resistance is a serious threat that occurs globally in the health sector due to increased consumption of inappropriate antibiotics. Guidelines for prescribing antibiotics for ARTIs have been issued in general practice to promote rational antibiotic prescribing. This study was conducted to compare the effectiveness of cefixime and tetracycline as a solution to improve monitoring of appropriate antibiotic use in the treatment of ARTIs. All stock isolates were rejuvenated first, and cultured on standard media and Kirby–Bauer disc diffusion method was used for susceptibility testing in accordance with the Clinical and Laboratory Standard Institute’s (CLSI) recommendations. Identification of bacteria from a single isolate was carried out to determine which bacteria were resistant to cefixime and tetracycline. A total of 466 single isolates of bacteria were analyzed, which showed a percentage of resistance to cefixime 38.0%, and tetracycline 92.86%. Bacterial isolates were resistant to cefixime and tetracycilne was a genus of Haemophilus, Streptococcus, Corynebacterium, Staphylococcus, and bordetella. Cefixime compared to tetracycline was proven to be superior in terms of the effectiveness of ARIs treatment.

## Introduction

Acute respiratory tract infections (ARTIs), which involve the upper or lower respiratory tract are among the most common problems in general medical practice in developing countries, including Indonesia. The use of antibiotics has become a mainstay in its treatment, but it is now recognized that the benefits are often accompanied by disadvantages (adverse drug reactions, ineffective financial costs, and antibiotic resistance) ^1^. Antibiotic resistance can occur naturally, or clinically, which is the ability of bacteria to resist the effects of antibiotics ^2^. Overuse of antibiotics can contribute to an increase in cases of antibiotic resistance. Antibiotic prescribing guidelines for ARTIs in general practice have been established to ensure proper and effective treatment ^3^. ARTIs are the mainly reason for prescribing antibiotics in adults, and often they are not properly prescribed ^4^. The presence and spread of antibiotic resistance has become an increasingly serious public health problem ^5^.

These resistant bacteria will disrupt the success of the treatment process, and the increased cost of treatment per individual will also result in the epidemic spread of antibiotic-resistant infections ^6^. The implementation of policies to limit the spread of resistant bacteria from patient to patient includes improving hospital hygiene, using vaccines, monitoring rational use of antibiotics, and using antibiotic combinations ^7^.

Cefixime is a third generation cephalosporin antibiotic with activity as a bacteride which is able to damage the bacterial cell wall by the mechanism of action through inhibition of penicillin binding proteins, and damage the peptidoglycone synthesis pathway. Cefixime is widely used in many countries because it has broad spectrum activity against all Gram positive and negative pathogenic bacteria and atypical organisms, e.g. mycoplasma and chlamydia ^8^.

Tetracycline antibiotics are widely known to have a broad spectrum of activity, act on a variety of Gram-positive and negative bacteria, spirochetes, obligate intracellular bacteria, and are also effective against protozoan parasites ^9^. Tetracycline treatment for symptoms of respiratory tract infections is an antibiotic of choice, especially for infections caused by the potential pathogens of *Haemophilus influenzae* and *Diplococcus pneumoniae*. Medical recommendations are also given to patients with chronic airway obstruction receiving antibiotics, and also if they show symptoms of acute infection ^10^.

The study reported by Ramdhani et al., regarding the management of ARTIs patients in a public health center of Tasikmalaya, Indonesia, described a fairly serious case of antibiotic resistance. The use of several antibiotics showed decreased effectiveness with a high resistance value, including amoxicillin (70.25%), levofloxacin (50.0%), ciprofloxacin (43.03%) ^11–13^.

This study was conducted to evaluate the comparative effectiveness of cefixime and tetracycline through antimicrobial susceptibility testing (AST) in the empirical treatment of ARTIs. In addition, this study was also to determine the genus of total clinical isolates of patients who had resistance to cefixime and tetracycline antibiotics. Tests were carried out using stock isolates from patients obtained from previous studies.

## Materials and methods

The test sample was a combined stock isolate from previous ARTIs research totaling 466 single bacterial isolates. This sample has been purified from bacterial contaminants, and rejuvenated.

The bacterial growth medium used was Mueller Hinton Agar (MHA) (Oxoid) with a concentration of 38 g/L, according to the guidelines from the CLSI ^14^.

Biochemical test materials for bacterial identification include Lactose (Merck), Mannose (Merck), Maltose (Merck), peptone (Oxoid), phenol red (Taylor), Kovac`s reagent (Bio-Rad), TSIA , methyl red (HiMEdia), α-naphthosl (Merck).

### Rejuvenation and purification of clinical isolates

The technique of rejuvenating clinical isolates was carried out using the scratch plate method. Clinical isolates from previous studies were rejuvenated on new MHA growth media, and incubated at 37 °C for 18 h. Colony morphology observations were carried out including color, colony structure, and different haemolytic and morphological characteristics ^15^.

### Preparation of test bacteria suspension

The test bacterial suspension was prepared by inoculating the bacterial colony into a sterile physiological NaCl solution. The turbidity of the bacterial suspension should be made equal to the standard turbidity of 0.5 Mc Farland solution ^16^.

### Antimicrobial susceptibility testing (AST)

The Kirby–Bauer disc diffusion test was used to determine the sensitivity of antibiotics to bacterial isolates ^17^. This test can evaluate the effectiveness of antibiotics that are already resistant or are still sensitive by measuring the diameter of the inhibition zone. This testing technique is based on the diffusion principle through antibiotic paper disks. This test was carried out with 3 repetitions. Determination of the resistance value of the tested bacteria to cefixime and tetracycline were carried out by comparing the diameter of the inhibition zone with the standard diameter of the resistance zone formed ^14^. The value of the resistance level category can be seen in Table 1.

**Table 1:** Categories of cefixime and tetracycline inhibition zone diameter.

Categories	Cefixime (mm)	Tetracycline (mm)
Resistant	≤ 17	≤ 11
Intermediates	18–20	12–14
Sensitives	≥ 21	≥ 15

### Identification and morphologic and biochemical characterization

The gram stain technique, with microscopic visualization at × 100 magnification was used to ascertain cellular morphology, and bacterial classification. After identifying the phenotype and cell colonies, a conventional biochemical test was carried out to determine the classification of the isolated bacteria according to the existing biochemical testing protocol ^18^. The conventional biochemical tests are: GS = Gram Staining; MT = Motility Test; MR = Methyl Red; SC = Simone Citrate; TSIA = Triple Sugar Iron Agar; OX = Oxidase; UR = Urease Test, XY = Xylose; CT = Catalase Test; VP = Voges-Proskauer; carbohydrate fermentation test (LAC = Lactose, MAN = Manose; MAL = Maltose; SAC = Saccharose); IND = Indole Test ^19^.

## Results and discussion

### Biochemical conventional identification

Bacterial isolates were obtained from patients who had been confirmed by a doctor indicating that they had suffered from respiratory tract infections. Statistically showed that the number of bacterial isolates consisted of the genus Staphylococcus (35%), Streptococcus (29%), Bordetella (15%), Hamophyllus (12%), and Corynobacterium (9%).

**Figure 1:**
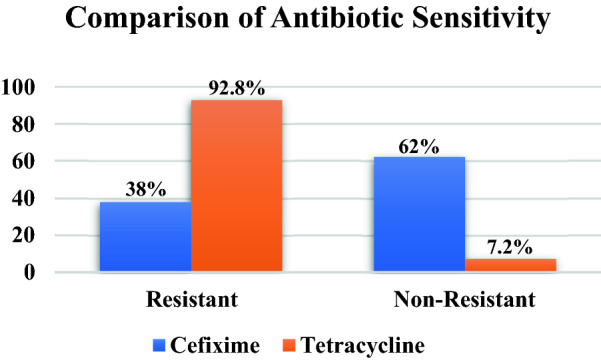
Total percentage of cefexime and tetracycline resistance test against bacterial isolates.

Biochemical identification were carried out on 466 stocks of bacterial isolates, identified as Gram-negative, Gram-positive, bacilli (rod-shaped), coccoid (rod-shaped), coccobacilli. Results analyzed bacteria, the biochemical conventional tests revealed 5 genus of bacteria: *Bordetella, Corynebacterium, Staphylococcus, Streptococcus, Haemophilus* (Table 2, Fig. 1).

**Table 2:** Results of biochemical test.

Group of bacteria	Shape	Gram	Genus	Biochemical test (positive)
1	Coccobacillus-capsulated	Negative	Bordetella	OX, CT,UR
2	Bacilli-rod shaped	Positive	Corynebacterium	MR, CT, TSIA, LAC, MAN, MAL
3	Round-shaped	Positive	Staphylococcus	CT, MR, VP, UR, SC, LAC, MAN, MAL
4	Coccus-capsulated	Positive	Streptococcus	LAC, MAL
5	Coccobacilli	Negative	Haemophilus	CT, OX, MAL

Several studies have shown similar results that of bacteria from the genus Bordetella, Haemophilus, Corynebacterium ^20–23^. Similar study conducted by Chopra et al. showed that *Streptococcus haemoliticus*, Staphylococcus, and *Corynebacterium diphtheriae* against tetracycline antibiotics ^9^. Cefexime is also reported to have started decreasing susceptibility patterns of these bacterial pathogens, Streptococcus pneumoniae, Haemophilus influenzae with low resistance levels. ^24^. Schico GC reported relevant information that cefexime showed decreased sensitivity to Staphylococcus, and streptococcus but was still very active than cefaclor and cefuroxime against Gram-negative respiratory pathogens ^25^.

However, some authors still highly recommend cefexime as a first-line antibiotic in overcoming cases of resistance to URTI and LRTI, especially against the pathogens *Streptococcus pneumonaie*, *Streptococcus pyogenes*, *Hemophylus influenza*, *Moraxella catarrhalis* ^26–28^. Other studies have also demonstrated good clinical efficacy data of cefexime in URTI and acute otitis media (AOM) cases, where community-induced infections exhibit high rates of resistance to macrolides and are highly sensitive to cefexime ^29,30^.

### Antibiotic susceptibility profile

The main focus of our study was to evaluate the current prevalence of bacteria responsible for ARTIs among the population in the urban area of Tasikmalaya, Indonesia. Our study investigated the correlation between pathogenic bacteria causing ARTIs and the antibiotic susceptibility profile commonly used by medical practitioners in public health centers. The test results obtained can be a comparative study regarding the effectiveness of cefixime and tetracycline as drugs of choice in the treatment of ARTIs. The disk antibiotic concentration used in accordance with CLSI guidelines are cefixime 5 µg, and tetracycline 30 µg^14^.

Antibiotic susceptibility test carried out on 466 bacterial isolate stocks showed that cefixime was superior to tetracyclines in the treatment of ARI. Cefixime showed a lower resistance level of 38.0% when compared to 92.86% tetracyclines. The level of tetracycline resistance was obtained from the number of bacteria genus Staphylococcus (158), Streptococcus (121), Bordetella (64), Hamophyllus (52), and Corynobacterium (37). In addition, the value of the cefixime resistance level was derived from the number of each bacterial genus Staphylococcus (69), Streptococcus (45), Bordetella (27), Hamophyllus (21), and Corynobacterium (15).

**Figure 2:**
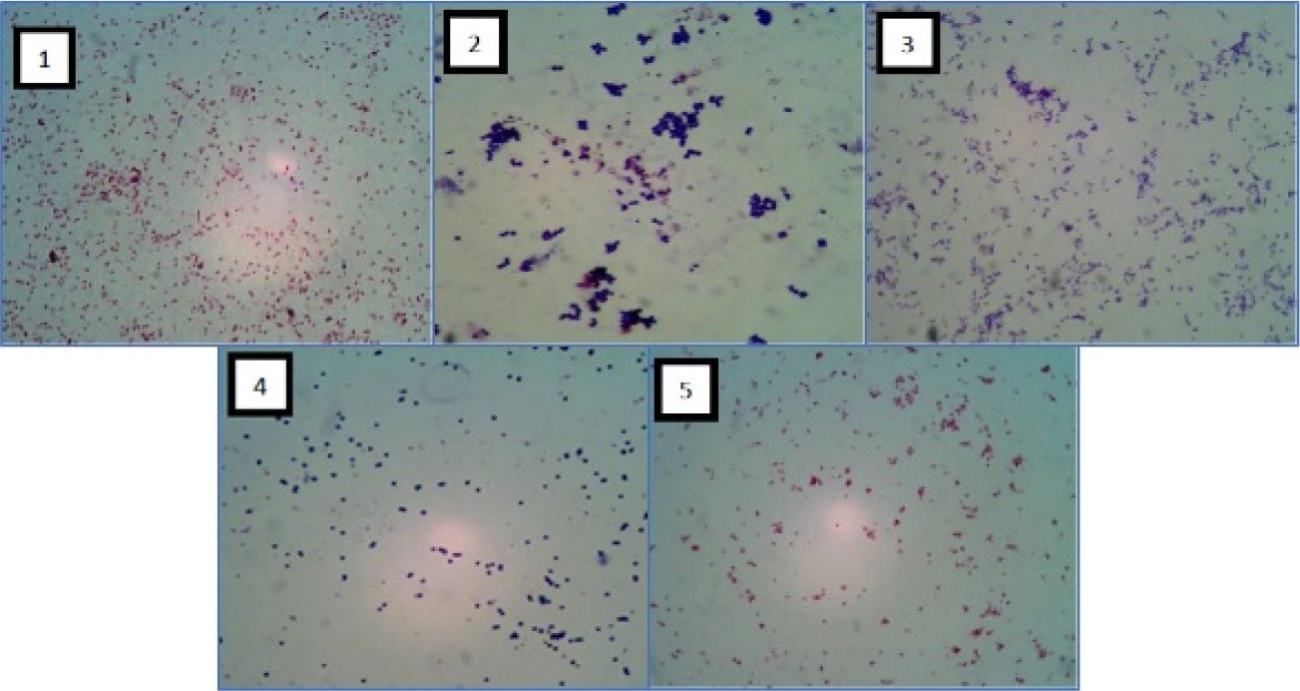
Gram stain of the bacterial group.

Additionally, the chi-square test was 308.0892, with the p-value was < 0.00001 and the result was significant at p < 0.05. These statistical data provide information for comparisons of resistant and non-resistant conditions from cefixime and tetracycline antibiotics described a significant difference in the level of resistance in the use of the two antibiotics for the treatment of ARTIs. These results are also consistent with other studies that cefexime is more effective in the treatment of respiratory infections compared to other drugs (eg ofloxacin, amoxicillin, and ciprofloxacin) ^24,31^. The susceptibility of categories of isolates of both antibiotics are shown in Fig. 2.

These results will provide important information to the Health Office of the City of Tasikmalaya to determine the antibiotic procurement policy for public health centers, and evaluate the pattern of prescribing antibiotics for ARTIs treatment by practicing doctors. Synergic cooperation and coordination between all health professions in the prevention of ARI is also very necessary.

Coordination with local policy holders of the Tasikmalaya City Health Office regarding the evaluation of the use of antibiotics in the treatment of ARTIs has been carried out. Preventive policies and education such as the intervention of antimicrobial stewardship programs (ASPs) at reducing unnecessary antibiotic prescribing, including training communication between health professionals, accountable justification of health programs, feedback by comparing socio-behavioral responses through questionnaires. Making handouts that are distributed to patients in community health centers as part of a program to educate the public about the use of appropriate antibiotics can support the success of ARTIs therapy. We plan to measure the impact of these interventions in the near future.

## Conclusions

Cefexime treatment has shown superior effectiveness compared to tetracyclines in the case of ARTIs. Cefexime can still be used as a front line antibiotic option in the management of ARTIs.

The study also reported the identification of pathogenic bacterial organisms causing ARTIs and antibiotic resistance in accordance with research reports from another bacterial group *Bordetella, Corynebacterium, Staphylococcus, Streptococcus, Haemophilus.*
